# Family Context Assessment in Middle Childhood: A Tool Supporting Social, Educational, and Public Health Interventions

**DOI:** 10.3390/ijerph18031094

**Published:** 2021-01-26

**Authors:** Florencia Barreto-Zarza, Manuel Sánchez de Miguel, Jesús Ibarluzea, Llúcia González-Safont, Marisa Rebagliato, Enrique B. Arranz-Freijo

**Affiliations:** 1 Faculty of Psychology, University of the Basque Country (UPV/EHU), 20018 San Sebastian, Spain; manu.sanchez@ehu.eus (M.S.d.M.); mambien3-san@euskadi.eus (J.I.); e.arranzfreijo@ehu.eus (E.B.A.-F.); 2 Biodonostia Health Research Institute, Group of Environmental Epidemiology and Child Development, 20014 San Sebastian, Spain; 3 Spanish Consortium for Research on Epidemiology and Public Health (CIBERESP), 28029 Madrid, Spain; gonzalez_llu@gva.es (L.G.-S.); rebaglia@med.uji.es (M.R.); 4 Epidemiology and Environmental Health Joint Research Unit, FISABIO -Universitat Jaume I-Universitat de València, 46020 Valencia, Spain; 5 Predepartamental Unit of Medicine, Universitat Jaume I, 12071 Castellón de la Plana, Spain

**Keywords:** family context, positive parenting, assessment, validation, middle childhood

## Abstract

Quality of the family context has an important role in the physical and mental health of children; that is why it is important to have reliable and updated tools. This study aims to design and validate a new tool, the Haezi Etxadi Family Assessment Scale 7–11 (HEFAS 7–11), to assess family context quality in middle childhood. A sample of two cohorts of 772 Spanish families with children aged between 7 and 11 (M = 9.39 years; SD = 1.57; 51.2% girls), participated in the study. Results showed good psychometric properties for the instrument and the confirmatory factor analysis showed a five individual subscales structure: 1. Promotion of Cognitive and Linguistic Development (α = 0.79); 2. Promotion of Socio Emotional Development (α = 0.83); 3. Organization of Physical Environment and Social Context (α = 0.73); 4. Parental Stress & Conflict (α = 0.75); and 5. Parental Profile Fostering Child Development (α = 0.80). The association between HEFAS 7–11 and Trial Making Test was also analyzed to determine the concurrent validity of the instrument. The new scale shows its potential in the fields of research, social and educational, to know those variables that need to be promoted under the approach of positive parenting from a public health perspective.

## 1. Introduction

After multiple empirical studies carried out in the past decades, the importance that quality of family context has on children’s psychological development has been clearly established. Adverse environments, the lack of a structured context, and the low quality of parent–child interactions among others, can disrupt brain architecture and impair the development of higher-order cognitive functions such as executive functions and self-regulation [1,2]. Middle childhood is a key stage of development for maturing of the prefrontal cortex, the brain area involved in the development of these skills [3,4]. As such, having a family context measurement instrument would enable high-quality research to be conducted on the influence of this environment on these extremely important processes. Moreover, having up-to-date instruments that allow a comprehensive assessment, beyond the traditional evaluations focused only on parenting styles [5], is vital to understand the complexity of the multiple variables which make up any family context. The aim of this work is to construct a new instrument for the exhaustive assessment of the quality of the family context, focused on the developmental period between 7 and 11 years.

The framework most commonly used for classifying family context variables is that offered by Bronfenbrenner’s bioecological model [6,7], which sees development as the result of an interactive process between the individual, as the carrier of individual characteristics, and the contextual factors located at different system levels. This model also posits that an individual’s development is influenced by his or her immediate surroundings, such as the family within the microsystem, and that these contexts are in turn affected by factors in the broader environment, such as parents’ socioeconomic status or education level [8]. Following this theoretical model, we reviewed the extant scientific literature on family context variables that influence children’s neuropsychological development.

Starting with an analysis of the microsystem variables that foster cognitive and linguistic development, one element that is particularly worth mentioning is the quality of learning interactions based on the *Scaffolding* process. This term, which was coined by Wood, Bruner, and Ross [9], is defined as a process in which the “expert” (in this case the parents) helps the child in accordance with their current level of ability, gradually withdrawing their aid as the learning process is consolidated. The ultimate aim of this process is to promote autonomy and enable children to solve their problems independently [10]. Research into parental scaffolding has found that this variable predicts executive functions, self-regulated behavior, linguistic development and academic success among others, during middle childhood [11,12]. Another family context variable which fosters cognitive and linguistic development is *Promoting Play*, which classic theories of development consider to be a critical factor for cognitive development and social competence [13,14]. Carlson and White [13] found that role play games helped children develop self-control and executive functions through the process of psychological distancing. Both the scaffolding process and the promotion of play require the presence of available materials that facilitate its practice; the *Presence of Learning Materials* at home is a variable included from the first versions of the HOME scale, a well-known traditional tool aimed to assess family context quality [15].

In relation to microsystem variables, which promote social and emotional development, research findings indicate that fostering *Emotional Expressiveness* helps children recognize emotional states which, in turn, has been linked to understanding the actions of others based on a correct interpretation of their mental attributions, which is known as Theory of Mind [16]. Understanding other people’s emotions and actions and the internalization of self-regulation strategies are cognitively demanding activities which are important for children to develop during the school-going years [17], that is when they become more independent from their caregivers and begin to function in broader contexts. Another variable which fosters a high-quality affective climate is *Optimal Frustration*, which provides children with small-scale challenges to help them make the cognitive leap which will prepare them to cope with possible stressful situations in other contexts [18]. The ability to tolerate frustration, which initially develops in the family context, may become the first link in the chain to acquiring resilience later on in life. Equally important is *Setting Limits*, which is a necessary complement to optimal frustration and constitutes an external reference in relation to which children adjust their expectations regarding what adults expect of them in normal family life [19].

Along with the variables mentioned above, it is also important to highlight the *Fostering of Autonomy and Self-esteem*, which through task setting and decision-making, seeks to help children internalize behavior regulation strategies and gain a sense of responsibility [20]. It is worth remembering that contexts which foster confidence, thereby indirectly engendering a feeling of being useful, help support the promotion of autonomy. At the other extreme, psychological control by parents and/or a failure to acknowledge the child’s achievement upon completing the task set, have a direct and detrimental effect on children’s self-assessment [21]. Similarly, parental sensitivity for demanding the appropriate degree of autonomy from their child, alongside the predominance of support and affection, are at the heart of the democratic parenting style, which in turn has been associated with many different variables, including self-regulation [2], the development of executive functions [22], and academic performance [23], among others.

Another microsystemic variable that is also worth considering is the *Quality of Sibling Relationships*, since it is associated in middle childhood with Theory of Mind, prosocial behavior, the development of resilience and scaffolding in learning activities [24,25]. It is therefore necessary to assess whether parents generate an atmosphere conducive to fostering good relationships between siblings, as an important developmental resource [26]. The father’s involvement in the childrearing process is another key variable. Research findings have highlighted the important impact of the father figure on both the development of executive functions and internalizing and externalizing problems [27,28]. Indeed, some authors agree that the importance of the father has been underestimated, arguing that this variable should be included in both the designs of research studies usually carried out with mothers and in the establishment of political measures to promote better father–child relationships [29,30].

Also within the microsystem, a group of individual parental variables should be taken into consideration, as far as they are clearly influencing the quality of the interactions inside the family. This group includes *Parental Self-efficacy*, understood as parents’ perceptions regarding their own ability to bring up their children. As reported, for instance, in a study by Glatz and Buchanan [31] with a sample of children with a mean age of 11, recent research has shown that positive parental self-efficacy is associated with good parenting practices during childhood and adolescence. Individual exosystem variables also include *Implicit Theories* that refer to parents’ perceptions of the explanatory factors of child development, which is related to parental locus control. Those parents with an external locus of control, related to a developmental genetics perspective, would tend to be less involved in rearing which can affect child development [32,33]. It has therefore been included in this study to determine in future studies whether parents have an environmentalist or geneticist outlook, with a view to analyzing how this may affect their children’s development. Another variable to take into consideration is *Parents’ Knowledge of Child Development*, which they need to be aware of in order to adjust their demands to their child’s level of competence and not ask too much or too little of them at each developmental stage. Suskind et al. [34] have developed an instrument for assessing this variable, as a means of guiding families toward more appropriate practices for stimulating cognitive and emotional development.

In order to complete an in depth approach to family microsystem is relevant highlighting the impact that the presence of high parental stress and conflict have on daily family interactions clearly diminishing its quality. Regarding *Parental Stress*, several studies have shown that children’s exposure to what is described as toxic stress is a risk factor for their development [35,36]. A significant variable closely related to parental stress is the *Frequency of and Exposure to Conflict*, which is located mid-way between the individual variables and those of the parental subsystem within the microsystem. Past studies have identified exposure to parental conflict as a risk factor for healthy psychological development [37]. Levels of stress and conflict can be described as a type of proximal context inside the microsystem and conceptualized as a continuum, ranging from healthy and adaptive levels to toxic levels that negatively affects the quality family interactions and thereby children´s psychological development.

In relation to the exosystem, defined as a package of external social interactions, surrounding and influencing family interactions, the first variable to be mentioned is *Social Support Networks*; this variable refers to the quantity and quality of the relationships between the family system and the extended family and broader networks of friends and services. Some authors, such as McPherson et al. [38], group relationships within the family and community under a single concept called Social Capital, and highlight its protective role for child development. Finally, *Quality of the Physical Environment* and *Diversity of Experiences* are also included in this group. Both these variables stem from the instrument traditionally used to carry out an assessment of families in risk contexts, the above mentioned HOME scale [15].

Closely linked to the exosystem, there are some variables grouped in the so called mesosystem, which encompasses interactions between microsystems. Research has identified the quality of the *Family–School Relationship* as a variable that influences children’s mental health, social and emotional development, and academic performance [39]. Since both the family and the school are socialization contexts responsible for children’s upbringing and education, and given the long hours children spend in the latter, it is important to ensure a good quality relationship between the two in order to foster mutual support and collaboration strategies [40]. Another variable in this category is the Promotion of the Child’s Social Relationships. In a recent study by Finch, Garcia, Sulik, and Obradović [41] with a sample of 806 children aged between 8 and 10, the authors found that a greater degree of interaction with classmates was associated with better results in executive function evaluation tests. It should be remembered that peer relations are an important resource for both learning and socialization, particularly in a period in which children start to become increasingly independent of the family context [42].

Once the literature has been reviewed, and the main objective of this study being the elaboration of a new proposal for the evaluation of the family context focused on families with children between 7 and 11 years old, a grouping of variables to explore different areas of the family context is proposed. An accurate assessment of these areas would allow, on the one hand, to advance in the investigation on the relations between family context and psychological development in this particular developmental period. On the other hand, it would show data that would provide empirical support to the design of individualized family interventions and the implementation of evidence-based positive parenting programs, aimed at stabilizing the strengths of each family and promoting its weakest areas. The availability of an instrument to comprehensively assess the quality of the family context has important practical implications for professionals who work in the educational, social, and health fields, and specifically, in the public health area, given that the detection of protective and risk factors will allow the design of well-grounded interventions targeting the appearance of mental health problems, school failure, etc.

Based on previous factorizations of instruments to assess the quality of the family context in the developmental periods of 2 and 4 years [43,44], 5 assessment areas are identified to start the factoring process. The first two areas are grouping the variables promoting cognitive, linguistic, and socio-emotional development, these variables being genuinely microsystemic. The third area grouping the variables related to the quality of the physical and social context, these variables belonging to the exo and meso system. Given the great influence of variables related to stress and conflict on intra family interactive quality, it is proposed that they would constitute area number 4. In area number 5, are included those microsystemic variables related to the parental individual characteristics identified by literature as especially influential on family interactive quality. It is especially difficult to properly place the variable related to the father’s involvement, which location it is considered between the individual variables and those of the social context. Finally, it has been tentatively included in area 3, pending the analytical results.

Finally, it should be noted that the field of assessing the quality of the family context is currently a fruitful field of research, given its applied potential in the clinical, social, and educational fields. Among the relevant instruments, it is worth mentioning the North Carolina Family Assessment Scale [45], the Interview for the Assessment of Parental Competencies [46], the Family Care Indicators [47], the AIRE instrument that is a new adaptation of the HOME scale [48,49], and a very interesting exploratory large-scale family context assessment in early childhood developed by Niklas et al. [50]. In order to highlight this study contribution to the current knowledge on the field, it is considered that there is no validated instrument for the assessment of the family context in Spanish-speaking population, which gathers all the variables in the literature review, grouped in the same assessment protocol, and specifically focused in the 7–11 year developmental period. The highly accurate matching between the family context variables and developmental milestones in this period is also considered as a distinctive characteristic of the Haezi-Etxadi Family Assessment Scale 7–11 (HEFAS 7–11). With all this, the objective of the present study is to develop and validate a new protocol for the evaluation of the quality of family context in Spanish-speaking families, focused on the developmental period between 7 and 11 years.

## 2. Materials and Methods

### 2.1. Study Participants

Participants were recruited as part of the INMA (Infancia y Medio Ambiente-Environment and Childhood) Project, a Spanish multi-center population-based cohort study which aims to analyze the influence of environmental exposures and psychosocial factors on child health and development [51]. The data from two cohorts were used in this study: the Gipuzkoa cohort (a province located in the Autonomous Community of the Basque Country) and the Valencia cohort (from the Autonomous Community of Valencia). Pregnant women were recruited during their first prenatal visit (10–13 weeks gestation) to the reference hospital in each area (Zumárraga Hospital in Gipuzkoa and La Fe Hospital in Valencia). From 2014–2016, during the 7–8-year follow-up period in Gipuzkoa, 390 families completed a family context assessment. During the same period, 382 families completed the same assessment as part of the 10–11-year follow-up in Valencia. A total of 772 families with children aged between 7 and 11 (51.2% girls) therefore participated in the present study. All participants gave their informed consent during each phase, and the procedure was approved by the Ethics Review Boards at San Sebastian Hospital in the Basque Country and La Fe Hospital in Valencia.

### 2.2. Measures and Instruments

*The Haezi-Etxadi Family Assessment Scale 7–11 (HEFAS 7–11):* This instrument is a self-report questionnaire to assess the quality of family context, with a 6-point Likert-type response scale, which includes variables identified by recent literature as influencing development among school-aged children (7–11 years). The scale should ideally be completed jointly by the mother and the father, or the principal caregiver, always in the presence of a professional familiar with the instrument, who can clear up any doubts that may arise. The initial structure proposed prior to the factorization of the instrument is described below. This structure included 140 items distributed across five individual subscales. Completion time was approximately 25–30 min. Prior to the factor analysis, the variables were grouped as follows:

Subscale 1. Promotion of Cognitive and Linguistic Development (PCLD). 22 items distributed across four factors: 1.1 Presence of Learning Materials (PLM); 1.2 Promoting Play (PP); 1.3 Cognitive Scaffolding (CS); and 1.4 Linguistic Scaffolding (LS).

Subscale 2. Promotion of Social and Emotional Development (PSED). 31 items distributed across four factors: 2.1 Emotional Expressiveness (EE), 2.2 Setting of Limits and Optimal Frustration (SLOF), 2.3 Fostering Autonomy and Self-esteem (FAS) and 2.4 Quality of Sibling Relations (QSR).

Subscale 3. Organization of the Physical Environment and Social Context (OPESC). 46 items distributed across six factors: 3.1 Quality of the Physical Environment (QPE), 3.2 Social Support Networks (SSN), 3.3 Promotion of Child’s Social Relationships (PCSR), 3.4 Relations with the School (RS), 3.5 Diversity of Experiences (DE) and 3.6 Involvement of the Father or Secondary Reference Figure (FI).

Subscale 4. Parental Stress and Conflict (PSC). 17 items distributed across three factors: 4.1 Low Parental Stress (LPS), 4.2 Low Frequency of and Exposure to Parental Conflict (LFEPC) and 4.3 Conflict Resolution (CR).

Subscale 5. Parental Profile Fostering Child Development (PPFCD). 24 items distributed across two factors: 4.1 Parental Self-efficacy and Cross-cutting Competences (PSCC); and 4.2 Implicit Theories and Knowledge of Psychological Development (ITKPD).

*Trail Making Test (TMT)* [52]. This is a widely used test to evaluate the executive functions. In TMT part A, children are required to link a sequence of 25 numbered circles in ascending order; in TMT part B, participants had to connect numbers alternating between circles and numbered squares. In both cases, subjects were instructed to link the numbers as quickly as possible. For this study, only the response time in seconds of part B was used since it is more sensitive to cognitive flexibility and executive function [53]. Since these cognitive skills are very sensitive to interactive and contextual variables in the family context, we used the scores in Part B to determine the concurrent validity of HEFAS 7–11.

*Socio-demographic Questionnaire.* A general questionnaire designed ad hoc for the INMA Project was used to gather socio-demographic data. Information was collected on families’ social class (according to the Spanish National Classification of Occupations, CON-94), family structure, parents’ educational level, age, country of origin, child´s sex, number of siblings, and birth order. See Table 1.

### 2.3. Procedure

*Construction of the Haezi-Etxadi Family Assessment Scale 7–11 (HEFAS 7–11):* This instrument is based on its previous versions for children aged 2 [43,54] and 4 years [44]. First, a theoretical review was conducted of extant literature on family context assessment, with special focus on those family variables believed to influence psychological development during middle childhood. Based on the findings of this review, a total of 110 items were proposed for assessing the quality of family context among school-aged children. Subsequently, a team of five professionals working in the field of family psychology and development assessed the degree to which the items measured the different constructs proposed, using a 4-point Likert-type scale (1 = it is not relevant, 2 = it needs serious revision, 3 = it is relevant but needs minor revision, 4 = it is quite relevant). The scores were then used to calculate the content validity index (CVI), which was 0.81 for the 110 items. Similarly, 30 items were selected from the previous version of the Haezi-Etxadi Scale for 4-year-olds. These items were considered relevant to ensuring a high-quality family context during any stage of child development. They were related to parental stress, exposure to conflict, and quality of physical environment. The CVI for these 30 items was 0.95. Finally, the improvements suggested by the experts were carried out. The final version of the HEFAS 7–11, therefore comprised 140 items with a 6-point Likert-type response scale. To control for acquiescence bias, 34 of the 140 items were inverted.

*Face Validity:* Once the consensus-based version of the instrument had been obtained, a “Face Validity” test was conducted with 30 families of varying socioeconomic levels from the provinces of Valencia and Gipuzkoa. Participants were asked to rate the items from 1 to 10 for ease of comprehension and degree of correspondence with a 6-point response scale. They were also asked to highlight anything they did not understand and any suggestions for improvement. This qualitative analysis enabled possible problems to be identified in relation to how items are understood and interpreted. Next, the necessary changes were made to the instrument and a factor analysis was carried out using the final 140-item version.

*Data collection:* Participating families were contacted by telephone to arrange appointments at their local health center (in the case of the Gipuzkoa cohort) or the Faculty of Psychology (in the case of the Valencia cohort). The research team also requested that, if possible, both the father and the mother, or where appropriate the principal caregiver, attend the meeting. Participants were first given a brief set of instructions regarding the instrument and then completed the scale in the presence of the interviewer, who was there to help resolve any doubts. Children´s executive function was assessed following a strict protocol to minimise any measurement errors; the instructions were always given in the same order, a single trained evaluator was assigned to each child and there was sufficient space in the room between participants (maximum 2 at a time) to prevent any distraction.

### 2.4. Statistical Analyses

The statistical analysis of the data was conducted using SPSS 24 and AMOS 24 (IBM-SPSS Statistics). First, a descriptive analysis of the sample was carried out and the differences between the two cohorts were analyzed using the Student´s *t*-test and the Chi-square test, as appropriate in each case. Subsequently, in order to analyze the factor structure of the scale, the sample was divided into two on the basis of origin (Gipuzkoa or Valencia). With the data from the Gipuzkoa cohort (*n* = 390), an exploratory factor analysis (EFA) was conducted to determine the dimensionality of the five subscales which make up the proposed factor solution. Barlett’s sphericity tests were performed and the sampling adequacy was calculated using the Kaiser-Meyer-Okin (KMO) index. The factor analysis was conducted by extracting the principal axes and varimax rotation for each of the five subscales.

Next step was to confirm the EFA solution with the second half of the sample, being the Valencia cohort (*n* = 382). To this end, a confirmatory factor analysis (CFA) was conducted. The goodness-of-fit indexes were: the Comparative Fit Index (CFI), the Tucker-Lewis Index (TLI), and the Incremental Fit Index (IFI); values of >0.90 indicate a good fit. In addition, since chi-square (χ2) index is particularly sensitive to sample size, some authors [55] also recommend to take into account the ratio between chi squared and degrees of freedom (χ2/*df*); values under 2 on this test indicate a good fit, while values under 5 are considered acceptable. RMSEA (root mean square error of approximation) was also calculated; values lower than 0.05 indicate a good fit and those between 0.05 and 0.08 indicate a reasonable fit [56].

Subsequently, the descriptive analysis of the factorized HEFAS (7–11) was carried out. Weighted scores were calculated for each family context variable, with 16.67 being the lowest and 100 the highest possible score. Similarly, internal consistency indexes were calculated using the Cronbach’s Alpha coefficient. Finally, in order to determine the concurrent validity of the HEFAS, a Pearson correlation analysis was conducted between the five subscales and the Trail Making Test.

## 3. Results

### 3.1. Participants’ Sociodemographic Profile

Regarding the characteristics of the total sample, the mean age of participating mothers at testing was 41.38 years (SD = 3.84) and that of fathers was 43.41 years (SD = 4.66); both groups were mainly of Spanish origin (96% of mothers and 93.9% of fathers). Women had a higher education level than men, with 43.1% of mothers having university degrees, as opposed to 25.4% of fathers. Children had a mean age of 9.39 years (SD = 1.57), 51.2% were girls and 17% were singletons. Moreover, 57% were first-born children, 37.5% were second-born, 5% third-born, and 5% fourth or fifth-born. Finally, as regards family structure, the majority (85.8%) were traditional nuclear families (mother, father and child or children); 12.4% were single-parent families and 1.8% were step families. Socio-demographic data of the two cohorts of the study are also shown in Table 1.

### 3.2. Exploratory Factor Analysis

The exploratory factor analysis (EFA) with varimax rotation was carried out on the first half of the sample, belonging to the Gipuzkoa cohort (*n* = 390). In order to orthogonally test the maximum possible number of interactions, EFAs were carried out separately for each of the five subscales. Barlett’s sphericity tests were statistically significant for each subscale: Promotion of Cognitive and Linguistic Development (χ2 = 1874.73 *d.f*. = 66, *p* < 0.001), Promotion of Social and Emotional Development (χ2 = 8164.99 *d.f*. = 300, *p* < 0.001), Organization of the Physical Environment and Social Context (χ2 = 3680.913 *d.f*. = 231, *p* < 0.001), Parental Stress and Conflict (χ2 = 3372.506 *d.f.* = 105, *p* < 0.001), and Parental Profile Fostering Child Development (χ2 = 5395.564 *d.f.* = 276, *p* < 0.001). Thus, the hypothesis that the correlation matrix was in fact an identity-based one could be dismissed. The KMO index revealed optimum values of 0.85, 0.90, 0.78, 0.79, and 0.84, respectively. This enabled us to continue with the factor analysis through the extraction of the principal axes and varimax rotation. Those items that saturated at under 0.30, as well as those that saturated on two or more factors, were removed. As a result, of the 140 items initially proposed for the five subscales, 45 were eliminated.

The first subscale, Promotion of Cognitive and Linguistic Development, had a 3-factor solution which explained 51.38% of the variance. The Potential for Play factor was eliminated and the following constructs remained: Presence of Learning Materials, Cognitive and Linguistic Scaffolding and a new factor called Encouraging Reading. The second, subscale Promotion of Social and Emotional Development, presented a 5-factor solution explaining 57.40% of the variance. The constructs with which it was related were: Emotional Expressiveness, Setting of Limits and Optimal Frustration, Fostering Autonomy and Self-esteem, Quality of Sibling Relations and a new factor called Precedents of Self-regulated Learning. The third subscale, Organization of the Physical Environment and Social Context, presented a 4-factor solution explaining 43.70% of the variance. It was associated with the following constructs: Quality of the Physical Environment, Social Support Networks, Promotion of Child’s Social Relationships and Relations with the School (RS). In this case, the factor Diversity of Experiences was eliminated from the scale and Involvement of the Father or Secondary Reference Figure (FI) was moved to subscale 5.

The fourth subscale, Parental Stress and Conflict, presented a 3-factor solution explaining 50.13% of the variance. It was associated with the following constructs: Low Parental Stress, Low Frequency of and Exposure to Parental Conflict, and Conflict Resolution. The fifth and last subscale, Parental Profile Fostering Child Development, presented a 5-factor solution explaining 53.40% of the variance. In this case, four constructs were identified: Parental Self-efficacy, Knowledge of Psychological Development, Assertiveness and Environmentalist Outlook on Development. Finally, the factor Involvement of the Father or Secondary Reference Figure (initially proposed in subscale 3) was added here also.

### 3.3. Confirmatory Factor Analysis

To confirm the exploratory factor solution for each subscale of the HEFAS (7–11), a confirmatory factor analysis (CFA) was conducted with the families in the second half of the total sample, belonging to the Valencia cohort (*n* = 382). To explore the goodness of fit of the five subscales and their corresponding factors, we tried to replicate the factor solutions obtained in the previous EFA. Factor loadings were analyzed and we eliminated those items that failed to load significantly on the subscales´ factors. A total of 10 items were removed; therefore, 85 items made up the 5 HEFAS subscales. As shown in Table 2, the models of the five subscales were all statistically significant and had good fit indexes.

### 3.4. Descriptive Statistics and Reliability Analysis

Table 3 shows the descriptive analyses (mean and standard deviation) of the factorized HEFAS (7–11), as well as the minimum and maximum values obtained in each of the five subscales. It also shows the Cronbach’s alpha index with values of over 0.73 for the total of each of the five subscales, thus indicating a good level of reliability for the variables.

### 3.5. Concurrent Validity

In order to examine the predictive validity of the new scale, we analyzed the ability of the HEFAS 7–11 to predict children executive function using Pearson’s correlation. All five subscales of the HEFAS (7–11) are associated statistically significant in negative to executive function of children as measured by the TMT: Promotion of Cognitive and Linguistic Development (*r* = 0.237; *p* < 0.001)*,* Promotion of Social and Emotional Development (*r* = −0.318; *p* < 0.001), Organization of the Physical Environment and Social Context (*r* = −0.144; *p* < 0.001)*,* Parental Stress and Conflict (*r* = −0.123; *p* < 0.001) and Parental Profile Fostering Child Development (*r* = −0.147; *p* < 0.001). The factors for which no statistically significant association was found with the executive function measure were: Encouraging Reading, Social Support Network, Frequency of and Exposure to Conflict, Environmentalist Outlook on Development and Involvement of the Father or Secondary Reference Figure.

## 4. Discussion

In general terms, the results indicate that the main goal of designing and validating a questionnaire for assessing the quality of family context among children aged between 7 and 11 years was achieved. Therefore, this study offers a new updated instrument for evaluating the family context in a Spanish-speaking population, which includes all those variables identified by the literature as influencing psychological development during middle childhood.

If we analyze the different subscales starting with subscale 1, Promotion of Cognitive and Linguistic Development, we see that one factor from the initial proposal (Promotion of Play) was not confirmed by the factor analysis. This may be because the items included in the questionnaire were not discriminatory enough to reflect the variability in promotion of play among the families in the sample. Another explanation may be the absence of items designed to measure play using electronic devices, which are a common part of children’s play during this period. Another change observed was the merging of the two initial scaffolding factors into a single construct called Cognitive and Linguistic Scaffolding. Alongside the appearance of a new factor called Encouraging Reading, this seems to indicate that, during this developmental period, it may be more appropriate to describe development stimulation in terms of promoting learning rather than in terms of Scaffolding, a concept which implies a direct interactive process between parents and their child. Support for this hypothesis can be found in the factor Presence of Learning Materials, which not only remained after the CFA but was also found to explain the highest percentage of variance (31.6%). This factor implies the availability of materials and access to sources of stimulation which children use autonomously, under adult supervision. Finally, it is worth noting that the emergence of the factor Encouraging Reading attaches value to an activity which is closely linked to the development of executive functions and attention processes during middle childhood [57].

The results for subscale 2, Promotion of Social and Emotional Development, support the proposal outlined in the introduction section regarding the development of a more exhaustive measure than the traditional one focused on parenting styles [5]. The classic components of parenting styles, emotional warmth, and normative demands, are reflected in the factors Emotional Expressiveness, Fostering Autonomy and Self-esteem and Setting of Limits and Optimal Frustration. Moreover, the factor Emotional Expressiveness adds an important nuance linked to parental support for children’s emotion regulation. The soundness of the factor Quality of Sibling Relations is also worth remarking on, since both the EFA and the CFA confirmed the complete theoretical proposal of the items, and this factor was also found to explain the highest percentage of variance (23.5%) in this subscale. Quality of Sibling Relations assesses parents’ management of the relationships established between siblings in order to positively foster psychological development, the importance of which has been confirmed by recent empirical studies [24,58]. Finally, a new factor, which emerged as a result of the factor analysis, called Precedents of Self-regulated Learning, was difficult to categorize from a theoretical perspective, since it included items linked to the promotion of self-esteem, children’s freedom to express negative judgments and parental support for goal setting.

As regards the results for subscale 3, Organization of the Physical Environment and Social Context, the CFA confirmed the existence of four of the six factors included in the initial theoretical proposal. These four factors can be categorized according to Bronfenbrenner´s ecological systems theory [6,7]. Specifically, Quality of the Physical Environment and Social Support Networks are located in the exosystem ring, whereas Promotion of Child’s Social Relationships and Relations with the School are clearly located in the mesosystem, since they include interactions between different microsystems, such as peer group and school. It is worth highlighting that the highest percentage of explained variance (19.44%) was observed for Relations with the School, a finding which supports the inclusion of this aspect in family context assessments conducted from an ecological perspective. As regards the factors that were not confirmed, the absence of Diversity of Experiences, which explored parents’ efforts to expose their child to new experiences, may be due (as with Potential for Play) to the low discriminatory quality of the items. Finally, the Involvement of the Father or Secondary Reference Figure factor was moved to subscale 5, which is discussed below.

In subscale 4, Parental Stress and Conflict, the results confirmed the initially proposed theoretical model which includes the factors: Low Parental Stress, Low Frequency of and Exposure to Conflict and Conflict Resolution. The reliability indexes of the first two factors (α = 0.71 and 0.81), the level of variance explained (15.60% and 25.3%) support the soundness of the variables, despite their susceptibility to social desirability bias due to their intimate and private nature. The presence of stress and conflict in the family context can be categorized more precisely if their levels are placed on a continuum. This continuum runs from the positive end, which would include adaptive stress and constructive coping with conflict, to the negative end, which would include children’s exposure to destructive conflict and sustained exposure to negative stress. These are toxic situations that inhibit high-quality family interactions within the interactive systems which foster healthy psychological development. Recent studies show that the relationship between exposure to family conflict and the presence of adverse experiences is related to the presence of emotional and behavioral problems in children and adolescents [59,60].

Finally, in subscale 5 called Parental Profile Fostering Child Development, the factor structure proposed was not confirmed by the factor analysis. Nevertheless, the qualitative components of this subscale remained intact, with family context aspects which had previously been grouped together, simply being divided into separate factors. Thus, the new factor, Assertiveness, was established, which originally formed part of Parental Self-efficacy and Cross-cutting Competences; Parental Self-efficacy emerged as an independent factor. In addition, Knowledge of Psychological Development emerged as separate from Environmentalist Outlook on Development. Finally, Involvement of the Father or Secondary Reference Figure, previously located in subscale 3, wholly confirmed its initial set of items, with a good reliability index (α = 0.80) and a higher percentage of variable explained (21.3%) than the other factors in the subscale. This variable has been much better conceptually placed after the factorial analysis, being this way an important component of the parental profile which fosters child development. The inclusion of these variables renders the proposal for a more exhaustive analysis of family context quality even more comprehensive than the previous versions of the scale for earlier developmental periods [54]. According to current epigenetic approach to understanding psychological development [61], subscales 4 and 5, assess the individual parental variables which affect the type of parent–child bidirectional interactions that take place inside the microsystem, and which also influence other variables involved in phenotypical expressions of diverse areas of psychological development.

As regards the quality of the instrument presented, it is worth highlighting that in the predictive validity analyses, the scores for all subscales were significantly associated executive function scores from the Trail Making Test. In addition to attesting to the technical quality of the instrument, this finding indicates that the data obtained provides evidence-supporting association of high quality of the family context and less time in performing the TMT task, which indicates better executive function. This is an important fact, precisely because of the high sensitivity to environmental and interactive variables within the family context [62]. Although this evidence is still only correlational, but is important data since these skills form the cognitive infrastructure of self-regulation, which has been related to physical and mental health [63]. We hope to analyze this association in future studies with more robust analyses.

Another aspect to bear in mind when assessing the results of this study is that the data provided by the scale may be extremely useful for the correct design of individual interventions with both at-risk and non-at-risk families. This is in line with the Council of Europe Recommendation on positive parenting, in relation to the implementation of programs and initiatives to support parenting [64]. In relation to the design of preventive family intervention policies, the instrument may help detect trends and childrearing patterns in large-scale populations, allowing to know those variables of the family context susceptible to be included in the intervention programs. As mentioned by Perks and Cluver [65], a *parenting vaccine* in the form of parental support would contribute to reducing situations of violence, consumption of alcohol and other drugs, and mental health disorders, among others, problems that represent a serious threat to public health.

Some limitations should also be mentioned. First, although the five subscales all had good internal consistency indexes with values of between 0.73 and 0.83, a more detailed analysis revealed poorer reliability indexes for some factors. Specifically, the factors with a low Cronbach’s alpha were: Precedents of Self-regulated Learning (α = 0.58), Quality of the Physical Environment (α = 0.50), Conflict Resolution (α = 0.55), Knowledge of Psychological Development (α = 0.50), and Environmentalist Outlook on Development (α = 50). Future studies may wish to develop a more precise set of items to assess these constructs. Moreover, the instrument could be further improved by including a variable evaluating pattern of the time spent on and exposure to screens, since recent findings have shown this to be linked to a greater risk of obesity, sleep disorders, attention problems and poorer development of self-control [66]. Just as importantly, the scale could be enriched by the inclusion of a variable measuring the quality of family education in gender equality. Coeducation in the heart of the family is considered a prevention measure for all manifestations of gender-based violence: psychological, physical, and/or sexual [67].

Another important limitation was the use of a self-report measure, which is always subject to social desirability bias. This characteristic was partially compensated for by access to a large and socio-demographically diverse sample, which enriched the possibilities for analysis, particularly in the framework of a cohort which enabled access to longitudinal designs. Precisely, some statistically significant differences in the descriptive characteristics of both cohorts have been found. On the one hand, this could be a limitation in the analysis procedure when carrying out the CFA on a different sample. For this reason, in a future study we intend to analyze the invariance of the measurement to provide a new statistical property of the instrument. However, in the process of developing a scale, it is necessarily recommended in the literature to carry out the EFA and the CFA with two different data sets [68]. According to Curran and Hussong [69], when several samples are combined, the psychometric properties of one instrument are stronger, as it is possible to generalize the results to a more representative sample of the population. Another limitation of the study was the impossibility of testing a single model with all subscales. Following the recommendation of the literature, a ratio of the sample size to the parameters of 20:1 or at least 10:1 should be estimated [70]. Therefore, the sample size of this study led us to propose and test five different models, one for each subscale or area to be evaluated. For this reason, another issue to be considered is the need for a future study with a larger sample to test a single model that includes the grouping of the five subscales with all the items. In this way, the authors of this work understand that it is necessary to specify that this could be a preliminary study in which the statistical analyses conducted would be part of a broader validation process that is intended to be carried out in the future.

Finally, despite the aforementioned limitations, it is important to underscore that the family context assessment instrument obtained as a result of the EFA and CFA processes constitutes a reliable and exhaustive measure, which fills a gap and enables family assessment during middle childhood in a Spanish-speaking population. Specifically, this developmental stage is characterized by intense maturing and a high degree of plasticity [71]. As such, during this stage children are extremely susceptible to the influence of the diverse contexts in which their psychological development takes place, particularly the family context. Reliable knowledge and assessment of family context variables will therefore enable researchers to identify their contribution to interactions which, being internalized by children, help construct the final phenotype of each individual developmental process. In sum, the ultimate aim is to identify those interactions that influence the interactive epigenetic process, which in turn constitutes the current theoretical approach for conceptualizing human psychological development.

## 5. Conclusions

In this study a new instrument, the Haezi Etxadi Family Assessment Scale 7–11, is provided for the evaluation of family contexts in middle childhood in a Spanish-speaking population. This period is important for the development of cognitive and emotional skills, which are very sensitive to environmental variables and to social interactions in the family context. That is why it is important to have reliable and updated tools. The new HEFAS 7–11 presents adequate psychometric properties and includes those variables identified in the literature as influencing the development in school-age children. This new tool is useful in research and in the applied field, specifically in the social, educational, and public health fields, since it allows us to identify those variables of the family context, susceptible of being included in intervention programs for the promotion of children’s psychological development. The instrument also offers a useful tool for daily professional practice. The proper assessment of the family context can provide data that complete the necessary diagnosis in situations of family and educational orientation; also in judicial expert opinion processes, and in situations of psychopathological diagnosis with a systemic approach, among others. Our hope is to have contributed to the consideration of the positive parenting research field as a relevant public health issue.

## Figures and Tables

**Table 1:** Socio-demographic characteristics of the families participating in the study. Gipuzkoa and Valencia cohorts of the INMA Project.

	**Mother**					**Father**		
	**Total Sample**	**Gipuzkoa**	**Valencia**	***p*-Value**	**Total Sample**	**Gipuzkoa**	**Valencia**	***p*-Value**
**Age** Mean (SD)	41.38 (3.84)	40.30 (3.21)	42.48 (4.13)	<0.001	43.41 (4.66)	42.78 (4.41)	44.06 (4.82)	<0.001
**Education level**	**% (*n*)**	**% (*n*)**	**% (*n*)**		**% (*n*)**	**% (*n*)**	**% (*n*)**	
Primary	17.4 (134)	10.3 (40)	24.6 (94)	<0.001	30.7 (237)	20.8 (81)	40.8 (156)	<0.001
Secondary	39.4 (304)	36.2 (141)	42.7 (163)		43.1 (333)	47.9 (187)	38.2 (146)	
University	43.1 (333)	53.3 (208)	32.7 (125)		25.4 (196)	30.3 (118)	20.5 (78)	
Social class *								
High (I-II)	27.3 (211)	33.3 (130)	21.2 (81)	<0.001	24 (185)	28.7 (112)	19.1 (73)	0.002
Medium (III)	29 (224)	29.7 (116)	28.3 (108)		17.2 (133)	13.6 (53)	20.9 (80)	
Low (IV-V)	43.5 (336)	36.9 (144)	50.5 (193)		58.4 (451)	57.2 (223)	59.7 (228)	
**Country of origin**								
Spain	96 (741)	97.4 (380)	94.5 (361)	0.038	93.9 (725)	98.5 (384)	89.3 (341)	<0.001
Others	4 (31)	2.6 (10)	5.5 (21)		6.1 (47)	1.5 (6)	10.7 (41)	
	**Total Sample Children**		**Gipuzkoa Cohort Children**		**Valencia Cohort Children**			***p*-value**
**Age** Mean (SD)	9.39 (1.57)		7.88 (0.12)		10.98 (0.29)			<0.001
**Sex**	**% (*n*)**		**% (*n*)**		**% (*n*)**			
Girls	51.2 (396)		50.8 (198		51.8 (198)			0.768
Boys	48.8 (376)		49.2 (192)		48.2 (184)			
**Siblings**								
0	17 (131)		9.2 (36)		24.5 (95)			<0.001
1	66.5 (513)		72.3 (282)		60.5 (231)			
≥2	16.5 (128)		18.5 (72)		14.7 (56)			
**Birth Order**								
First	57 (439)		57.7 (225)		56 (214)			0.369
Second	37.5 (290)		37.9 (148)		37.2 (142)			
Third	5 (38)		3.6 (14)		6.3 (24)			
Fourth	0.5 (5)		0.8 (3)		0.5 (2)			
**Family structure**								
Traditional	85.8 (662)		93.8 (366)		77.5 (296)			<0.001
Single-parent	12.4 (96)		4.9 (19)		20.2 (77)			
Step-family	1.8 (14)		1.3 (5)		2.4 (9)			

Note. * Classified according to CNO-94. National Classifications of Occupations (Clasificación Nacional de Ocupaciones. España). Differences were tested by Student´s *t*-test and Chi square test.

**Table 2:** Confirmatory factor analysis fit indexes for the five HEFAS subscales.

HEFAS (7–11)	Factor Solution	χ^2^	*d.f.*	*p*	χ^2^/*d.f*	RMSEA	IFI	TLI	CFI
Subscale 1. PCLD	3-factors model	73.408	41	0.001	1.790	0.046	0.965	0.943	0.964
Subscale 2. PDSE	5-factors model	383.513	199	0.000	1.927	0.050	0.948	0.933	0.947
Subscale 3. OFESC	4-factors model	205.559	113	0.000	1.819	0.047	0.931	0.904	0.929
Subscale 4. PSC	3-factors model	96.380	49	0.000	1.967	0.050	0.964	0.941	0.963
Subscale 5. PPFCD	5-factors-model	291.793	176	0.000	1.658	0.042	0.936	0.913	0.933

Note. PCLD = Promotion of Cognitive and Linguistic Development; PDSE = Promotion of Social and Emotional Development; OEFCS = Organization of the Physical Environment and Social Context; PSC = Parental Stress and Conflict; PPFCD = Parental Profile Fostering Child Development.

**Table 3:** Descriptive statistics, Cronbach’s Alpha coefficient, and correlations through Pearson’s coefficient (r) between the factored HEFAS (7–11) and the executive function variable evaluated with the TMT.

Haezi Etxadi Family Assessment Scale (HEFAS 7–11)	Media	SD	Alpha de Cronbach	TMT
Subscale 1. Promotion of Cognitive and Linguistic Development (SCLD)	70.18	12.82	0.79	−0.237 **
Presence of Learning Materials (PLM)	69.49	15.88	0.62	−0.294 **
Cognitive and Linguistic Scaffolding (CLS)	71.30	15.25	0.72	−0.201 **
Encouraging Reading (ER)	69.50	18.92	0.70	−0.065
Subscale 2. Promotion of Social and Emotional Development (PSED)	82.45	8.59	0.83	−0.318 **
Emotional Expressiveness (EE)	93.99	8.75	0.64	−0.212 **
Setting of Limits and Optimal Frustration (SLOF)	83.52	11.42	0.70	−0.289 **
Fostering Autonomy and Self-esteem (FAS)	78.90	13.87	0.61	−0.313 **
Precedents of Self-regulated Learning (PSRL)	87.41	10.63	0.58	−0.251 **
Quality of Sibling Relations (QSR)	76.41	12.37	0.62	−0.096 *
Subscale 3. Organization of the Physical Environment and Social Context (OPESC)	88.11	7.34	0.73	−0.144 **
Quality of the Physical Environment (QPE)	93.78	9.31	0.50	−0.196 **
Social Support Networks (SSN)	89.18	10.53	0.70	0.002
Promotion of Child’s Social Relationships (PCSR)	73.50	17.56	0.77	−0.181 **
Relations with the School (RS)	92.13	10.21	0.65	−0.102 **
Subscale 4. Parental Stress and Conflict (PSC)	77.82	10.17	0.75	−0.123 **
Low Parental Stress (LPS)	71.14	16.68	0.71	−0.084 *
Low Frequency of and Exposure to Conflict (LFEC)	84.30	9.70	0.81	−0.044
Conflict Resolution(CR)	77.44	18.29	0.55	−0.174 **
Subscale 5. Parental Profile Fostering Child Development (PPFCD)	80.17	9.28	0.80	0.147 **
Self-efficacy (SE)	77.50	17.28	0.77	−0.173 **
Knowledge of Psychological Development (KD)	83.09	12.01	0.54	−0.297 **
Assertiveness (As)	88.78	10.71	0.60	−0.091 *
Environmentalist Outlook on Development (EOD)	78.68	17.79	0.54	−0.037
Involvement of the Father or Second Reference Figure (IFSRF)	75.93	14.94	0.80	−0.028

Note. SD = Standard Deviation; TMT = Trail Making Test; ** *p* < 0.01; * *p* < 0.05.

## Data Availability

The data presented in this study are available on request from the corresponding author. The data are not publicly available due to the privacy of the information provided by participants with respect to the specific educational, social and family context.
